# Development of a quantitative polymerase chain reaction assay and environmental DNA sampling methods for Giant Gartersnake (*Thamnophis gigas*)

**DOI:** 10.1371/journal.pone.0222493

**Published:** 2019-09-16

**Authors:** Gregg Schumer, Eric C. Hansen, Paul J. Anders, Scott M. Blankenship

**Affiliations:** 1 Cramer Fish Sciences-Genidaqs, West Sacramento, CA, United States of America; 2 Eric Hansen Consulting, Sacramento, CA, United States of America; 3 Cramer Fish Sciences, Moscow, ID, United States of America; University of Helsinki, FINLAND

## Abstract

The Giant Gartersnake (*Thamnophis gigas*) is a low density visually evasive species with a low detection probability based on standard field survey methods (e.g., traps, visual census). Habitat loss has resulted in extirpations or serious declines for *T*. *gigas* populations throughout the southern two thirds of its historic range. Uncertainty regarding its current distribution and occupancy present management challenges for the species. Enhancing survey sensitivity through development of environmental DNA sampling (eDNA) methods would improve compliance monitoring under the Endangered Species Act, recovery planning for *T*. *gigas*, and evaluation of California’s Central Valley tule marsh habitat on which this species depends. To address these needs, we designed and validated diagnostic quantitative Polymerase Chain Reaction (qPCR) assays for identifying portions of the Cytochrome B (CytB) and the Nicotinamide adenine dinucleotide (NADH) dehydrogenase subunit 4 (ND4) genes of the *T*. *gigas* mitochondrial genome. The designed ND4 qPCR assay was not specific to *T*. *gigas* DNA and amplified DNA from a closely related and spatially co-occurring *Thamnophis* species (*T*.*s*. *fitchi*). The CytB *T*. *gigas* qPCR assay proved specific to a species level with a sensitivity that reliably detected *T*. *gigas* DNA at a concentration of 2.0x10^-5^ ng μL^-1^. To assess detection range, coordinated field sampling was conducted at aquatic sites with an observed and documented population of *T*. *gigas*. The *T*. *gigas* qPCR assay reliably detected DNA from samples taken 300m downstream from the known source. We then used environmental eDNA sampling and qPCR analysis to augment unsuccessful trap surveys in the southern range of *T*. *gigas* and detected DNA in 28 of the 52 locations sampled, confirming that *T*. *gigas* was still present at some sites where physical trapping failed to identify presence. QPCR-based DNA detection coupled with eDNA sampling methods provides an effective means to obtain critical population metrics from this otherwise cryptic, federally protected and hard to study organism, offering great promise for elucidating patterns of occupancy with greater efficiency and at far less cost than trapping methods, particularly where detection probabilities are low.

## Introduction

The Giant Gartersnake (*Thamnophis gigas*) is a federally and state threatened species endemic to California’s Central Valley. Described as among California’s most aquatic gartersnakes [1], *T*. *gigas* is associated with low gradient streams, valley floor wetlands, and marshes. The species requires wetlands to forage for prey (i.e. fish and amphibians), upland areas for basking, upland burrows as summer shelter, and higher elevation uplands for winter brumation [2–5]. *T*. *gigas* typically emerges in March, is active (foraging and breeding) through spring and summer, and seeks winter refuge in the fall [5–9]. As a wetland species, *T*. *gigas* has been historically known to associate with marshes, ponds and low-gradient streams. The species is also associated with rice agriculture and the water supply channels supporting its practice [2,5,8,9]. Identifying when and where *T*. *gigas* occupy native habitat is a fundamental first step in any recovery action.

Land use practices have also negatively impacted *T*. *gigas*. An estimated 91% of California’s total wetlands has been lost since the 1780s due to agriculture and urban use conversions [10], with approximately 43% of freshwater wetlands in the Central Valley having been lost or converted since 1939[11]. While loss of historical habitat for *T*. *gigas* has resulted in extirpations or serious declines throughout the southern two thirds of its former range, additional threats may also contribute to the ongoing decline of the species [5,12]. Multiple threats may be particularly significant in the San Joaquin Valley (Southern Central Valley), where recent surveys indicated a rapid decrease in *T*. *gigas* abundance in areas where putative habitat remains [13–16]. Due to declines in abundance and spatial distribution, *T*. *gigas* was listed as threatened by the U.S. government in 1993 [4] and the State of California (California Code of Regulations 1971).

*T*. *gigas* is a secretive and evasive species that likely occurs at low density in some locations. Any efforts to document the presence of the species in specific locations which, through time provides the ability to also assess the current distribution and occupancy of *T*. *gigas* must, therefore, include survey and analytical methods (e.g. [17,18]) that account for low expected detection probabilities. Monitoring efforts informing the recovery process are currently limited to visual encounter and aquatic trapping surveys [17,18] both of which are associated with low or imperfect rates of detection [18], potentially leading to false conclusions of absence [19]. Physical trapping relies on protocols that are both time and labor intensive [18] and often is hindered by theft and tampering in areas with public access, potentially biasing survey results and endangering the health of the animals present in the census population. For rare, cryptic species with low detection rates, accurate detection is of paramount importance. Falsely declaring a species absent from a site can lead to inappropriate conservation and management decisions, or at worst, extirpation of populations.

Environmental DNA methods provide a means to address limitations of visual and trapping surveys, because they are: 1) cost effective and feasible to deploy over a large survey area, 2) unambiguously identify target organisms, and 3) sensitive, being capable of detecting trace amounts of DNA in sampled material [20–22]. The eDNA approach differs from traditional sampling in that a given survey does not capture the target organisms themselves, but the biological material those organisms leave in their environment [23]. In order to implement surveys that seek to use DNA to detect species of interest, both a DNA barcode and the means to assay for target DNA must exist. DNA barcoding is a successful technique for identifying species using a short DNA sequence from a standard position in the mitochondrial genome. DNA barcode sequences are very short relative to the entire genome and presently exist for many organisms or can be created reasonably quickly using routine laboratory practices. The Cytochrome C Oxidase subunit 1 mitochondrial region (COI) has emerged as a standard barcode region [24], while both the CytB and ND4 mitochondrial regions have proven to be equally adept at identifying higher animals [25]. The promise of using standardized DNA sequences to unambiguously identify organisms has captured the attention of the scientific community, government agencies and the general public [24]. There has been much eDNA work on amphibians and reptiles in general however, there is no known peer reviewed work prior to this manuscript explaining qPCR assays or eDNA surveys for any species closely related and coexisting to *T*. *gigas*. Bringing the power of molecular biology (quantitative PCR of species-specific barcodes) and eDNA approaches to enhance *T*. *gigas* survey method sensitivity would improve compliance monitoring under the Endangered Species Act, recovery planning for *T*. *gigas* and evaluation of the wetland habitat on which this species depends [4,5].

## Materials and methods

### CytB DNA barcoding

**Fig 1:**
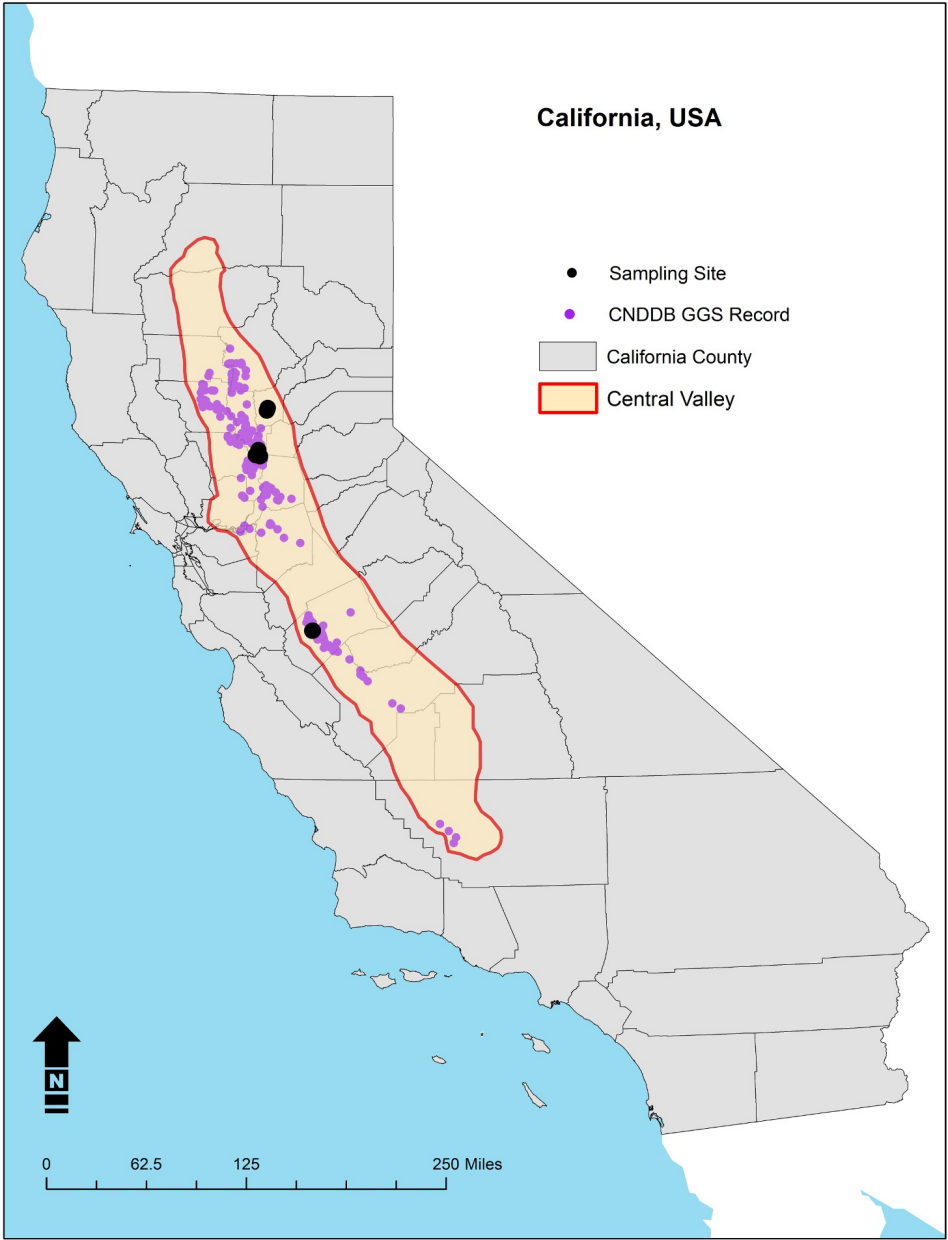
*T*. *gigas* tissue sample site location map. Location of the Central Valley within California, USA and locations of tissue samples collected for *T*. *gigas* quantitative polymerase chain reaction assay development.

Tail clips from five individually vouchered *T*. *gigas* were used as templates for DNA barcoding at the CytB and ND4 regions of the mitochondrial genome. Tail clips were sampled non-lethally as part of previous and ongoing studies. Tail clips ≤3mm in length (i.e., the minimum necessary to acquire live tissue) were collected without analgesic using sterilized surgical scissors during standard field processing, after which the tail tip was cleansed with betadine and sealed with surgical (cyanoacrylate) glue. Collected tail clips were preserved in 95% ethanol in the field and transferred on ice to the laboratory where they were then transferred to a -20°C freezer for long-term storage at Cramer Fish Sciences-Genidaqs, 3300 Industrial Blvd. Suite 100, West Sacramento, CA 95691, U.S.A. Other tissue samples collected from the same localities are archived at the U.S. Geological Survey Western Ecological Research Center, San Diego Field Station, 4165 Spruance Road, Suite 200, San Diego, CA 92101, U.S.A. In order to capture extant genetic diversity of the CytB gene within the *T*. *gigas* population, the five *T*. *gigas* specimens included wild individuals from across the species’ known range (Fig 1). DNA was extracted from each of the five individual tissues using Qiagen DNeasy® Blood and Tissue Kit (Qiagen, Inc.) following the manufacturers protocol. A 964 bp fragment of the CytB gene was amplified using the amphibian-specific PCR forward primer MVZ15-L GAACTAATGGCCCACACWWTACGNAA [26] and reverse primer CytbAR-H TAWAAGGGTCTTCTACTGGTTG [27]. Additionally, all sequence data for the mitochondrial ND4 gene specific to *T*. *gigas* was harvested from NCBI nucleotide data base. The ND4 sequence information was aligned in order to identify conserved regions within the species for the purpose of generating a consensus fragment unique to *T*.*gigas*. A BLAST (Basic Local Alignment Search Tool) was conducted on the ND4 consensus sequence. The consensus fragment was later used as template for qPCR assay design. The consensus fragment was generated in-silico, no PCR products were generated for ND4.PCR amplification for the CytB gene consisted of a 15 μl total reaction volume. Each 15 μl reaction was composed of 7.5 μl Promega GoTaq® G2 Hot Start Colorless Master Mix (Promega Corporation), 1 μl 10 nM Forward primer, 1 μl 10 nM Reverse primer, 3.5 μl ultra-pure nuclease free water and 2 μl 100 ng μL^-1^ normalized DNA. Thermocycling was performed using the Promega Master Mix protocol with an optimized annealing temperature of 55° C and the complete cycle profile of 2 min. at 95° C initial denaturation, 40 cycles of 95° C for 30 sec, 55° C for 30 sec, 72° C for 1 min, with a final extension at 72° C for 5 min. PCR products were separated by electrophoresis in 1% agarose (w/v) gel at 90v for 20 minutes. The gel was visualized by BioRad mini trans illuminator (BioRad Laboratories, Inc.). The appropriate bands were excised from the gel using a brand-new razor blade for each band and placed into individual sterile micro-centrifuge tubes. DNA was extracted from the agarose gel using QIAquick® Gel Extraction Kit following manufacturer’s guidelines. Extracted DNA along with forward and reverse sequencing primers were submitted to UC Davis DNA sequencing facility (Davis, CA.) for DNA Sanger sequencing. DNA sequence data received from UC Davis sequencing facility were aligned using Geneious alignment software (Geneious, Inc.) and analyzed for a lack of variability across the 964 bp regions. A consensus fragment was used as the template for a nucleotide BLAST.

### qPCR assay design, validation and optimization

#### Design

Consensus sequences for both the *T*. *gigas* mitochondrial genes CytB and ND4, or DNA barcodes, were used as templates for qPCR assay design. CytB and ND4 consensus sequences were sent to Integrated DNA Technologies (IDT) scientific applications support for qPCR assay design and incorporation of Locked Nucleic Acid base technology (Table 1). Assays specific to both the CytB and ND4 genes of *T*. *gigas* were identified by IDT and cross referenced against off target sequence data from closely related and co-existing congener species within the *Thamnophis* genus: *T*.*s*. *fitchi*, *T*. *couchii*, *T*. *elegans* and *T*. *atratus* for assay specificity.

**Table 1:** qPCR assay details. CytB primer and probe sequences used for Giant Gartersnake eDNA survey. PrimeTime® LNA® (Locked Nucleic Acid) base positions are indicated by (+).

Species	Oligo	Sequence 5’-3’	Reporter	Quencher
*Thamnophis gigas*	GGS CytB-F	ACAAACCTACTAACCGCCG		
*Thamnophis gigas*	GGS CytB-R	GGCAAAGAATCGTGTTAAGGTC		
*Thamnophis gigas*	GGS CytB Probe	CC+GA+G+A+TA+T+GGT	6 FAM	BHQ-1
*Thamnophis gigas*	GGS ND4-F	TTAAAACTAGGAGGCTACGGC		
*Thamnophis gigas*	GGS ND4-R	GGGCAAGGACGATAAATGGA		
*Thamnophis gigas*	GGS ND4 Probe	AA+C+C+CTC+CC	6-FAM	BHQ-1

#### Validation

The primer probe sets for CytB and ND4 were tested for specificity and cross reactivity in-vitro using DNA as a template from five vouchered specimens of *T*. *gigas* and two vouchered specimens from each of the closely related and co-existing congeners *T*.*s*. *fitchi*, *T*. *couchii*, *T*. *elegans* and *T*. *atratus*. The PCR for specificity was performed in triplicate in 5 μl total volume containing 1 μl 20 ng μL^-1^ of DNA template, 2.5 μl TaqMan Universal Master Mix (Thermo Fisher ABI), 0.5 μl each, 900 nM initial concentration of both forward and reverse primers and 1 μl 3.0 μM initial concentration probe. Primer and probe optimization were conducted following Applied Biosystems (Thermo Fisher ABI) guidelines for optimizing primer and probes for amplifying custom target sequences. PCR for optimization was performed in 5 μl total volume containing 1 μl of DNA template, 2.5 μl TaqMan Universal Master Mix (Thermo Fisher ABI), 0.5 μl each, 50-900nM final concentration of both forward and reverse primers, and 1 μl 50–250 nM final concentration probe (*T*. *gigas* CytB Probe). Thermocycling for the specificity and optimization PCR reactions were conducted on a BioRad CFX96 (BioRad) with the following cycle conditions: initial activation 10 min at 95° C followed by 40 cycles of 15 sec denaturation at 95° C and 1 min extension at 60° C. All PCR reactions were conducted with three no template controls run in parallel. Resulting data were analyzed using the BioRad CFX manager 3.1(Bio-Rad Laboratories, Inc.). To determine the sensitivity of the assay, tenfold serial dilutions ranging from 0.0000002 ng μL^-1^ to 2 ng μL^-1^ were amplified using the *T*. *gigas* assay. Each of the tenfold serial dilutions was amplified ten times along with four no template controls using the same cycle conditions as described for specificity and optimization.

### eDNA field sampling and sample analysis

**Fig 2:**
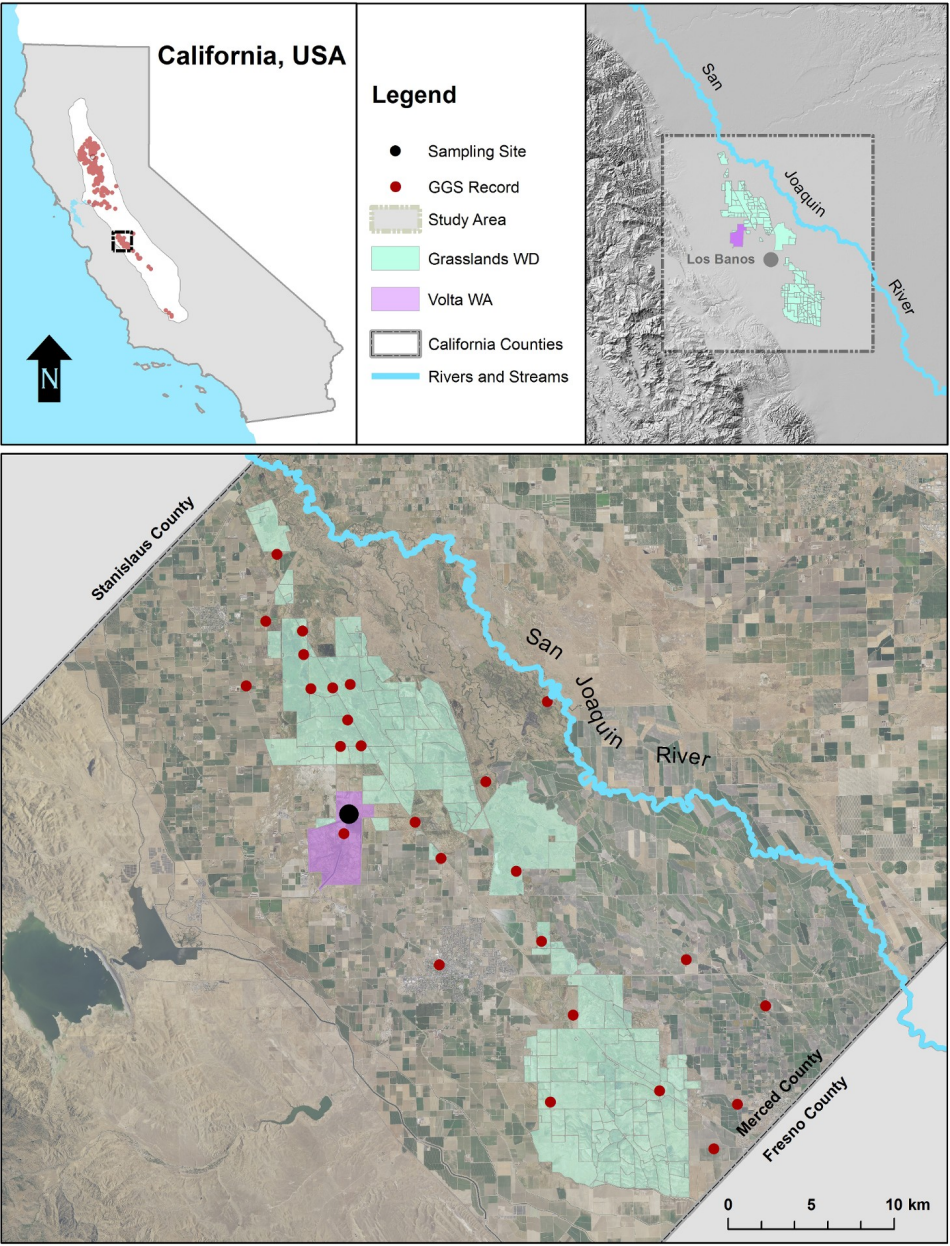
eDNA filed sample location. Location of the field site within California, USA (top left), location of the study area relative to the Volta Wildlife Area (top right), and the sampling sites (bottom). The black dots represent the sampling site, and the red dots represent historical *T*. *gigas* locality records.

**Fig 3:**
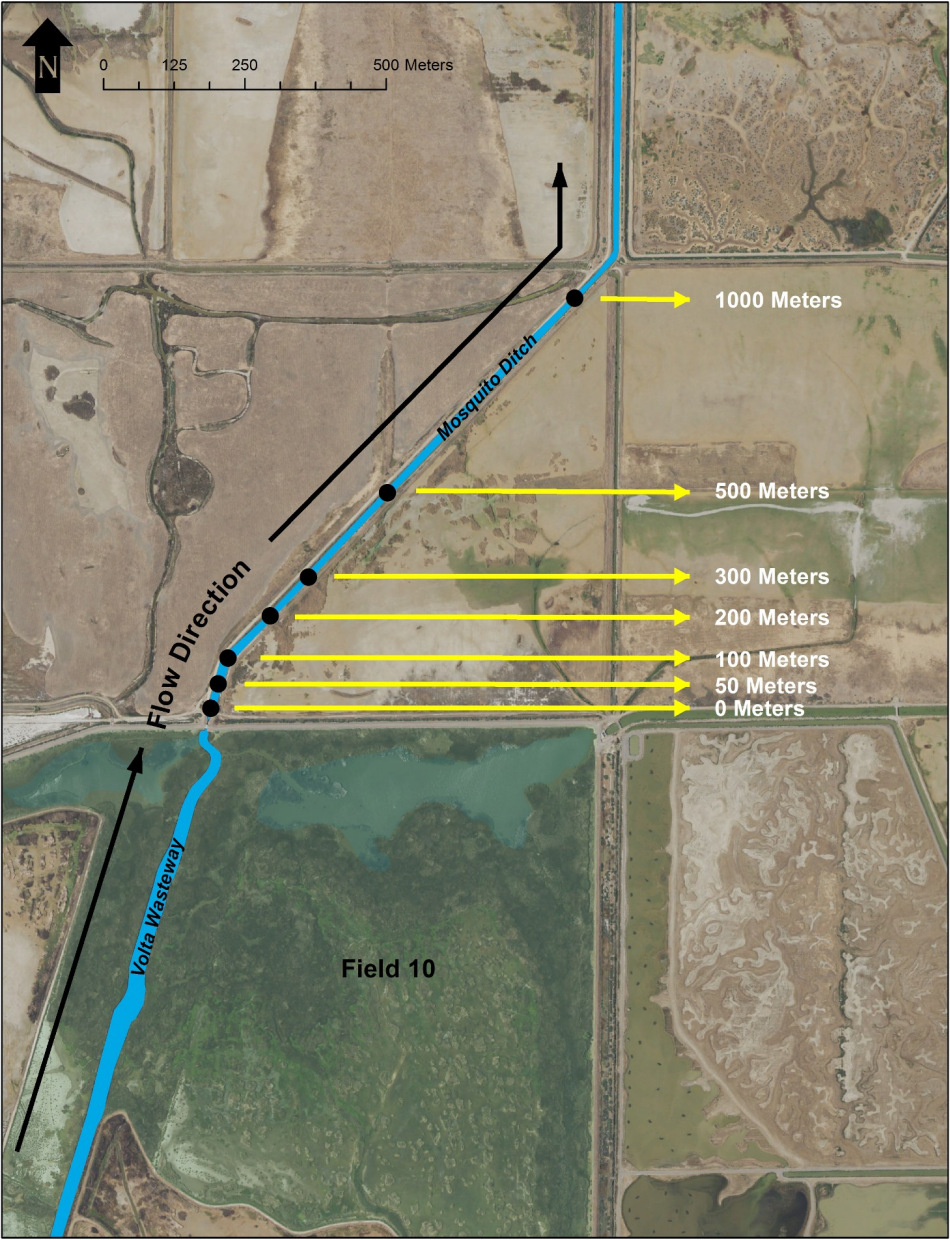
*T*. *gigas* eDNA validation sample sites. Location of the field validation sites on the Mosquito Ditch and their position relative to Field 10 on the Volta Wildlife Area and to one another.

Field samples were taken at pre-determined intervals from the Grassland Water District’s Mosquito Ditch, which receives flows via a screw gate immediately downstream of a verified *T*. *gigas* population in the Volta Wildlife Area’s Field 10 (Merced County, California; Figs 2 and 3). This site was selected due to the paucity of adjacent wetlands from which *T*. *gigas* might immigrate and its relative isolation from other occupied locales in the Grasslands Ecological Area ([28–30]; Fig 2). On August 11, 2016, single filter samples were taken within Mosquito Ditch starting downstream at 1000m and working upstream towards Field 10 on the Volta Wildlife Area at site 0m.

Field sampling and laboratory protocols followed procedures described in [23], with the two exceptions being that samples were taken aboard a kayak and Millipore Sterivex™-GP 0.45μm sterile filter unit (EMD Millipore) were used in place of Millipore Sterivex™-GP 0.22μm. We found that using Millipore Sterivex™-GP 0.45μm in place of the Millipore Sterivex™-GP 0.22μm increases the volume of water that can be filtered. Water samples were collected from either the bank margin, or where this was infeasible due to dense vegetation or steep topography, by kayak at the channel center. All samples taken from the kayak were taken from the upstream side only. For each sampling event, water was filtered directly from the water body at an approximate depth of 6–10 inches below the surface using sterile Saint Gobain XL-60 silicon tubing (Tygon®; internal diameter 6.3mm), and a portable Masterflex1 L/S Easy-Load II peristaltic pump (Cole-Parmer®) powered by a cordless hand drill. Water samples were filtered through a single Millipore Sterivex™-GP 0.45μm sterile filter unit filters clogged. No water was transported or stored during sampling nor was any water transported between sampling sites; instead all filtration occurred directly on the boat at each site. Sample filtrate was captured and measured in graduated flasks to verify the volume of each sample. Filtered water was then poured over the side of the boat after completion of sampling at each site. To ensure that field equipment was free of contamination, DNA field control samples were taken for each sampling day. Each field control consisted of Sterivex™ filtered ultra-pure water transported from the lab and processed in the same fashion as the field samples. The field controls were processed for the presence of *T*. *gigas* DNA in parallel with all other samples. To eliminate cross contamination between sites due to equipment or the investigator, sterile gloves and all sampling materials were pre-packaged in the laboratory and discarded after one use. Tubing and gloves were immediately disposed of after each use into a sealed trash bag on board. All filters were likewise considered single use. After filtration, filters were capped at each end, labelled with location ID, placed into a sterile secondary container, sealed, and immediately placed on ice. All filters were kept on ice in a cooler for the duration of the sampling day, after which they were transferred to a -20°C laboratory freezer. The filters were stored within individually sealed secondary containers at -20°C until DNA extraction.

DNA from all samples and controls was extracted using PowerWater Sterivex™ DNA Isolation Kit (Mo Bio Laboratories, Inc.) following the manufacturer’s recommended guidelines. A DNA extraction negative control was processed in parallel to ensure sample integrity throughout extraction procedure. The DNA extraction control consisted of Sterivex™ filtered ultrapure water only. DNA extraction controls were processed using the same equipment used to extract DNA from all samples. Each sample and all controls were analyzed in triplicate, with each qPCR technical replicate consisting of a 10 μl reaction volume, for the presence of the *T*. *gigas* DNA using the qPCR *T*. *gigas* primer and probe designed as part of this study. Each 10 μl qPCR reaction was composed of 2x Applied Biosystems TaqMan Universal PCR Master Mix, No AmpErase UNG (Thermo Fisher ABI), optimal primer probe concentrations (900 nM initial primer concentrations, 3 μM initial probe concentration for both ND4 and CytB and 4 μl DNA template. Thermocycling was performed using a Bio-Rad CFX 96 Real Time System (Bio-Rad Laboratories, Inc.) with the following profile: 10 min at 95° C, 40 cycles of 15 sec denaturation at 95° C and 1 min extension at 60° C. Six negative template control (NTC) reactions were run on the plate with the control sample templates consisting of 4 μl of ultrapure water replacing DNA template within reaction volume. Three positive control reactions consisting of 20 ng μL^-1^ *T*. *gigas* genomic DNA template were also tested in parallel to ensure consistent PCR performance. All PCR master mixes were made inside a UV PCR enclosed workstation. DNA template was added to the master mix outside of the UV PCR workstation on a dedicated PCR set up workbench. All PCR reactions were conducted on instruments located outside of the main lab in a separate portion of the building. Results of the qPCR reactions were analyzed using BioRad CFX manager v3.1 (Bio-Rad Laboratories, Inc.).

## Results

### DNA barcoding

DNA sequence data received from the UC Davis sequencing facility were aligned and analyzed for a lack of variability across 964 and 833 base pairs from within the CytB and ND4 regions of the *T*. *gigas* mitochondria genome respectively. Consensus fragments were 687 bp and 684 bp in length for CytB and ND4, respectively, having the least intra-species variability and the most inter-species variation. These consensus fragments were used as the template for a nucleotide BLAST. BLAST results evaluating the 687 bp consensus CytB fragment and the 684 bp ND4 fragment were evaluated by IDT scientific applications support team for qPCR assay design incorporating PrimeTime® LNA® (Locked Nucleic Acid) qPCR 5’ probes. At the time this experiment was conducted the IDT PrimeTime® LNA® probe design was a proprietary process. The IDT scientific applications support team provided results of the design algorithm only. There was neither an indication of what algorithm parameters were applied nor how the algorithm assigned LNA base pairs within the probe sequence. This was unique in that most qPCR assay design algorithms, proprietary or otherwise, have parameters that can be adjusted to meet user criteria. Resulting assays including PrimeTime® LNA® probes and associated forward and reverse primer pairs are listed in Table 2. The assays listed in Table 2 are exact sequences as received from IDT, including LNA® base pairs indicated by a + in front of associated nucleotide.

**Table 2:** Results of qPCR analysis for Giant Gartersnake DNA.

Sample	Results	Cq1	Cq2	Cq3	Avg Cq
**1000m**	-	ND	ND	ND	NA
**500m**	-	ND	ND	ND	NA
**300m**	+	36.27	36.68	36.73	36.56
**200m**	+	37.2	36.95	36.80	36.98
**100m**	+	35.51	35.65	34.28	35.14
**50m**	+	35.42	35.64	35.48	35.51
**0m**	+	35.10	34.92	34.78	34.93
**Negative Field Control**	-	ND	ND	ND	NA
**Extraction Control**	-	ND	ND	ND	NA
**No Template Control**	-	ND	ND	ND	NA
**Positive Control**	+	12.49	12.25	12.41	12.38

“+” = positive, “-”= negative, and ND = no detection at sample locations in Mosquito Ditch. Sample locations are labelled as meters from Field 10. Quantification cycle (Cq) is shown for each technical replicate of a positive test for *T*. *gigas* DNA and the average (Avg) Cq across all replicates.

### qPCR assay validation

#### Optimization, specificity, and sensitivity

Both the CytB and ND4 assays were found to perform optimally using an initial primer concentration of 900 nM for both the forward and reverse primers. The optimal probe concentration was determined to be 3 μM initial concentrations for both CytB and ND4. Optimized assay conditions were used to evaluate species specificity using two individuals each from *T*.*s*. *fitchi*, *T*. *couchii*, *T*. *elegans* and *T*. *atratus* in addition to the five vouchered *T*. *gigas* specimens. The DNA from all species in the cross-reactivity panel were normalized to 20 ng μL^-1^. However, both assays amplified *T*. *gigas* DNA normalized to 20 ng μL^-1^from all 5 vouchered specimens. however, the ND4 assay also cross reacted or amplified the DNA from both *T*. *elegans* and *T*. *s*. *fitchi*. Because ND4 was shown to be non-specific in detecting *T*. *gigas* DNA it was removed from consideration as a viable option to unambiguously detect *T*. *gigas* DNA and was not subjected to further validation. The CytB assay showed no cross reactivity with closely related and co-existing congeners with the exception of *T*. *couchii*. The conditional cross reactivity with *T*. *couchii* was deemed acceptable as the change in PCR conditions required for successful amplification of *T*. *couchii* DNA significantly diverted from PCR conditions to successfully amplify *T*. *gigas* DNA. In addition, the habitat of *T*. *gigas* and *T*. *couchii* has not been shown to overlap. Therefore, the potential for false positive detections of *T*. *gigas* by cross reacting with *T*. *couchii* was considered insignificant. The 2.0x10^-4^ ng μL^-1^ dilution was amplified in 100% of the replicates with an average C(q) of 33.85. The 2.0x10^-5^ ng μL^-1^ dilution was successfully amplified for 60% of the 10 replicates with and average C(q) of 36.3 Following the MIQE definition, the limit of detection (LOD) for quantifying target DNA with reasonable certainty (95% probability) is between 2.0x10^-5^ ng μL^-1^ and 2.0x10^-4^ ng μL^-1^ The standard curve for *T*. *gigas* produced the slope of -3.3222 suggesting a qPCR efficiency of 100%. Further, A Y-intercept of 40.661 indicated that the assay was sensitive, and 40.661 cycles is sufficient to detect any target DNA. An R^2^ value of 0.9974 revealed a high correlation between the C(q) and the concentration of template.

#### eDNA field samples

Samples collected from Mosquito Ditch were tested in triplicate for the presence of *T*. *gigas* DNA. Sample volumes were distinctly low given water conditions in Mosquito Ditch and varied from 20–50 ml/sample. A sample was considered positive for the presence of *T*. *gigas* DNA if any one of the three technical replicates showed logarithmic amplification within 40 cycles. The 1,000 meter and 500-meter samples showed no amplification of *T*. *gigas* DNA. The 300 to 0-meter samples all amplified *T*. *gigas* DNA with an average C(q) ranging from 34.98 to 37.78. Negative controls did not amplify *T*. *gigas* DNA and the positive control amplified *T*. *gigas* DNA with and average C(q) of 12.26 (Table 2).

## Discussion

*T*. *gigas* has experienced many presumed extirpations since its listing as a rare species in 1971 (e.g., [31,32]), particularly in the southern portion of its range [33]. A clear progression in sampling technique and detection rates has occurred as a result. For example, from the 1940s through the 1970s, *T*. *gigas* in the San Joaquin Valley occurred at densities facilitating routine observations through visual encounter surveys (e.g., [1,31]). Where subsequent visual encounter surveys later failed to produce comparable detections (e.g., [32,34], intensive trapping surveys continued to confirm presence at many sites (e.g., [13,29,33,35]). Despite adherence to rigorous trapping protocols [18] and improvements in trap construction designed to increase detection probability ([36]; E. Hansen unpublished data), detections have since diminished to the extent that *T*. *Gigas* is now presumed to be extirpated throughout much of the San Joaquin Valley (e.g., [16,29,31,32]). As part of a separate project conducted for the Fish and Wildlife Service in 2015 and 2016, the authors worked to ascertain the presence, distribution, and relative abundance of *T*. *gigas* in the San Joaquin Valley using physical trapping techniques, but also collected environmental DNA samples to augment trap survey results. Consistent with regional efforts conducted over the past decade, visual encounter and trap surveys failed to detect *T*. *gigas* in most areas despite the presence of putative habitat. Conversely, use of the eDNA sampling techniques described here in combination with qPCR analysis confirmed that *T*. *gigas* was still present at some sites where physical trapping failed to identify presence [19]. *T*. *gigas* DNA was detected in 28 of 52 locations sampled, indicating presence at 8 of the 17 trap sites as well as sites where trapping proved infeasible [19]. While these results did not provide detailed information regarding relative abundance because *T*. *gigas* were not physically captured, they can be used both to model occupancy and to guide future demographic and genetic studies.

Diminishing detection rates associated with intensive physical trapping throughout the San Joaquin Valley likely reflected continuing downward abundance trends for *T*. *gigas* subpopulations. It is worth noting, eDNA sampling techniques applied by the authors since developing the assays reported here, suggest that *T*. *gigas* may be more broadly distributed in the San Joaquin Valley than previously assumed, albeit at very low densities [19]. Surveys designed to document current species distribution and occupancy must, therefore, include sensitive survey and analytical methods that account for low expected detection probabilities [17–19]. If current distribution and occupancy information is unreliable, then efforts to obtain demographic information (e.g., survival, fecundity) and population genetic information are hindered and management actions will invariably be sub-optimal. QPCR-based DNA detection coupled with eDNA sampling methods provide a means to obtain critical population metrics from this otherwise cryptic and other hard to study organisms. Enhancing survey method sensitivity will also improve compliance monitoring under the Endangered Species Act, recovery planning for *T*. *gigas*, and evaluation of California’s Central Valley tule marsh habitat on which this species depends.

During the publication process, the authors became aware of an internal agency report by the U.S. Geologic Survey (USGS) evaluating eDNA techniques for use in monitoring *T*. *gigas* [37]. Given that the overarching intent of our work is broader access to enhanced monitoring capabilities, we felt there was value in considering all newly available information, despite the delay it caused to the publication process. Surprisingly, the USGS was unable to detect *T*. *gigas* DNA in either field trials or mesocosms that contained *T*. *gigas*. Therefore, the agency report provided no insights into improvements that could be incorporated into field sampling procedures. While we cannot speculate on the causes for non-detection, the authors are not aware of another species incapable of being detected via eDNA methods. The USGS report did reference a new proprietary commercial product that was of interest, PrimeTime® LNA® bases manufactured from IDT. We worked with the manufacturer to produce a version of our CytB qPCR assay that incorporated PrimeTime® LNA® bases We elected to adopt the assay version containing LNA® bases for publication. As noted above, the assay from the USGS report could not be recreated given the information provided, as the LNA® bases were not specified in their report. The manufacturer (IDT) assisted us with recreating a version of the USGS assay that contained PrimeTime® LNA® bases, but its exact similarity is unknown. The version of the USGS assay the we created did not outperform the CytB assay described in Table 2.

In conclusion, the new qPCR assay reported here, coupled with e-DNA sampling techniques, are encouraging developments for the conservation and management of the *T*. *gigas*. Our empirical study confirmed the value of this combined approach [19], which compared favorably to the even most recent conservation efforts for *T*. *gigas* [37]. While results and methods reported here will help improve our understanding of the contemporary occupancy of *T*. *gigas*—which in turn will facilitate the focused trap surveys required to evaluate the demographic and genetic status—this approach is also applicable to a wider array of taxa of conservation and management concern, particularly cryptic remnant species found at low densities with low detection probabilities in altered environments. While the techniques described here provide immediate value, further work evaluating the performance of eDNA sampling techniques remain beneficial. Furthermore, site selection is critical to the success of sampling surveys, which currently rely on local expertise. Describing field sampling best practices and relating those field procedures to inform future eDNA surveys will likely continue to improve our ability to provide critical information for successfully managing this imperiled taxon.
